# A commentary on ‘Bone grafting for femoral head necrosis in the past decade: a systematic review and network meta-analysis’ (*Int J Surg*. 2023;10.1097. doi: 10.1097/JS9.0000000000000231)

**DOI:** 10.1097/JS9.0000000000000416

**Published:** 2023-11-16

**Authors:** Jian Cao, Xufeng Wan, Qiang Su, Zongke Zhou

**Affiliations:** Department of Orthopedic Surgery, West China Hospital, Sichuan University, Chengdu, People’s Republic of China

*Dear Editor,*

We were highly interested in the article entitled ‘Bone grafting for femoral head necrosis in the past decade: a systematic review and network meta-analysis’ by Lou *et al*.^1^. The authors compared various bone grafting methods for patients with osteonecrosis of the femoral head (ONFH) who underwent core decompression (CD). Their findings suggest that bone grafting after CD can effectively prevent the progression of ONFH and that combining bone grafts with bone marrow grafts (BG+BM) appears to be more effective in preventing conversion to total hip arthroplasty (THA), slowing disease progression, and improving the Harris hip score (HHS). However, this article has raised some concerns for us.

Firstly, Lou *et al*. overlooked two eligible studies. Lakshminarayana *et al*.^2^ conducted a prospective longitudinal observational study, which compared the HHS and ONFH progress of patients with nontraumatic avascular necrosis treated by standard CD (36 hips) and CD with fibular grafting (40 hips). At the final follow-up (mean, 53.5 months), radiographic progression was observed in 25% of hips that underwent CD alone and in 30% of hips with fibular grafting, with median HHS values of 77 and 71.5, respectively. The authors concluded that CD with or without fibular grafting is effective for early-stage ONFH. Another missed retrospective cohort study by Ou *et al*.^3^ included 90 patients with ONFH at stage II with 122 hip joints. The study compared HHS and hip joint survival time between 60 hip joints treated with CD alone and 62 hip joints treated with CD and free fibular graft in combination. The authors concluded that CD combined with a free fibular graft can effectively improve hip joint function, relieve postoperative pain, and prolong hip joint survival time.

Inappropriate or inadequate search strategies may fail to comprehensively identify records that are included in bibliographic databases. The incomplete search of this meta-analysis may be due to an incomplete database search and an inappropriate search format. Firstly, the Cochrane Handbook^4^ states that the minimum search databases to be covered are CENTRAL, Embase, and Medline, but the authors only searched PubMed, ScienceDirect, and the Cochrane Library. Secondly, in order to include the most comprehensive literature possible, the authors should have added a good deal of free-text terms for each medical subject heading. However, the authors only used “femoral head necrosis” or “avascular necrosis of the femoral head” and “bone graft” as keywords. To further test our conjecture, we conducted our own search using medical subject headings (“femoral head necrosis” and “bone graft”) and related terms (“transplantation, bone” “femur head necrosis” “ischemic necrosis of femoral head” “avascular necrosis of femur head” “avascular necrosis of the femoral head” “osteonecrosis of the femoral head”) in the title or abstract. Our search revealed that the study by Lakshminarayana *et al*. was found in Medline, and the literature by Ou *et al*. was found in Embase. We emphasize the importance of a comprehensive search strategy in systematic reviews to minimize publication bias and provide a reliable estimate of effects.

After reading this paper online, we performed a retrospective study in our institution to compare the effectiveness of CD vs. CD+ABG (autologous bone graft) vs. CD+BBG (biomaterial bone graft) for the early nontraumatic ONFH. At a mean follow-up of 7.2 years, the improvement in HHS from preoperative to final follow-up was 11.0, 12.2 and 11.1, respectively, in the CD group, the CD+ABG group, and the CD+BBG group (*P*=0.232). In addition, the rate of conversion to THA was 49.4%, 42.5%, and 41.9% in the three groups (*P*=0.367) (Table 1). We concluded that bone grafting after CD can slightly improve the HHS and reduce the incidence of conversion to THA.

**Table 1:** Improvement in Harris hip score and incidence of conversion to total hip arthroplasty.

Characteristics	CD	CD+ABG	CD+BBG	*P*
Patients, *N* (%)	302 (72.9%)	90 (21.7%)	22 (5.3%)	/
Hips, *N* (%)	356 (72.2%)	106 (21.5%)	31 (6.3%)	/
Gender, *N* (%)				
Female	56 (18.5%)	11 (12.2%)	1 (4.5%)	/
Male	246 (81.5%)	79 (87.8%)	21 (95.4%)	/
Age, years	39.5±9.1	41.4±8.9	43.8±8.5	/
Harris hip score				
Preoperative	70.9±5.6	70.8±5.8	71.3±3.6	0.927
Postoperative	81.8±4.1	83.0±4.0	82.3±4.1	0.035^a^
Improvement	11.0±6.4	12.2±6.5	11.1±4.8	0.232
Conversion to THA, *N* (%)	176 (49.4%)	45 (42.5%)	13 (41.9%)	0.367

ABG, autologous bone graft; BBG, biomaterial bone graft; CD, core decompression; THA, total knee arthroplasty.

a The difference between the CD and CD+ABG groups in postoperative HHS was significant.

We greatly appreciate the important study by Lou *et al*. that examined the efficacy of different bone grafting methods for ONFH after CD. Performing bone grafting after CD is crucial to delay the progression of ONFH. In addition, bone grafts, in combination with bone marrow grafts, appear to be most effective in achieving symptomatic relief and slowing the progression of ONFH. However, we must use the findings of this paper with caution due to the potential for bias.

## Ethical approval

Not applicable.

## Consent

Not applicable.

## Sources of funding

Not applicable.

## Author contribution

J.C.: literature search and writing and X.W.: literature search and data collections.

## Conflicts of interest disclosure

There are no conflicts of interest.

## Research registration unique identifying number (UIN)

Not applicable.

## Guarantor

Zongke Zhou.

## Data availability statement

Public access to the database is closed. For us, all related datasets were permitted to access and use by the Clinical Trials and Biomedical Ethics Committee of West China Hospital, Sichuan University. And the datasets used and/or analyzed during the current study are available from the corresponding author on reasonable request.

## Provenance and peer review

Not invited.
